# A novel report on the use of an oncology zygomatic implant-retained maxillary obturator in a paediatric patient

**DOI:** 10.1186/s40729-017-0073-7

**Published:** 2017-03-28

**Authors:** Amit Dattani, David Richardson, Chris J. Butterworth

**Affiliations:** 1 Oral and Maxillofacial Surgery, Regional Maxillofacial Unit, University Hospital Aintree, Liverpool, UK; 2 Maxillofacial Surgery, Regional Craniofacial Unit, Alder Hey Children’s Hospital, Liverpool, UK; 3 Maxillofacial Prosthodontics, Regional Maxillofacial Unit, University Hospital Aintree, Longmoor Lane, Liverpool, L9 7AL UK

**Keywords:** Zygomatic Implant, Hemi-maxillectomy, Oncology implant, Maxillary obturator, Zygomatic fixtures, Myxoid spindle cell carcinoma

## Abstract

This report details the use of zygomatic oncology osseointegrated implants to support and retain a maxillary obturator in a 13-year-old male patient who underwent a right-sided hemi-maxillectomy (Brown Class 2b) (Brown and Shaw, Lancet Oncol 11:1001–8, 2010) for a myxoid spindle cell carcinoma. At the time of maxillary resection, two zygomatic oncology implants were inserted into the right zygomatic body and subsequently utilised to provide in-defect support and retention for a bar-retained maxillary acrylic obturator prosthesis, which restored the patient’s aesthetics and function to a very high level. Close follow-up over 2 years demonstrated ongoing excellent function and disease control with no deleterious effects on facial or dento-alveolar growth clinically. This is the first clinical report of its kind in the published literature detailing the use of a zygomatic implant-retained obturator in a paediatric patient.

## Background

Maxillary defects of acquired [1] or congenital origin produce a communication between the oral and nasal cavities sometimes via an opening into the maxillary antrum and by direct communication into the nose. This in turn can result in masticatory compromise, swallowing and speech impairment, nasal fluid regurgitation and aesthetic concerns. The management of the maxillectomy patient is a complex area where there is still much debate [2], but in the paediatric patient, there is virtually no literature detailing the most appropriate approach. The use of microvascular free-tissue transfer has gained in popularity over time in adults in order to effect a biological closure of the resulting oro-nasal communication, but in the paediatric patient with maxillary malignancy, the use of a prosthetic obturator is more commonly reported [3]. The use of free-tissue transfer in children in the maxillofacial region seems to be mainly restricted to reconstruction of the mandible from the reviewed available literature [4] presumably because prosthetic obturation can offer good results in the maxilla and defer additional complex surgeries to a later date.

**Fig. 1:**
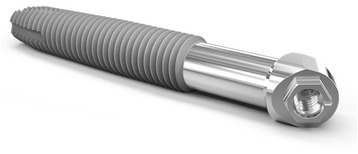
Zygomatic oncology implant with cleansable polished surface for intra-oral component

An obturator is a custom-made denture prosthesis that is used to close the communication with the antrum/nose in order to allow satisfactory mastication and speech. In the dentate patient, maxillary obturator prostheses may be retained by the natural dentition together with the engagement of undercuts within the maxillary defect itself. The use of osseointegrated implants to assist with the retention of a maxillary obturator has been reported [5]; utilising both dental implants into the residual alveolus and, more recently, the use of zygomatic implants in large maxillectomy defects has also been described [6]. Osseointegrated zygomatic implants provide rigid support and retention for the overlying implant-retained obturator with two, three [7] or four zygomatic implants being used for rehabilitation of a bilateral maxillectomy resection. There is no real information available on the use of zygomatic implants in the support and retention of obturator prostheses for unilateral maxillary defects in the dentate patient, but this situation mandates the use of two implants to allow splinting and bar construction to provide the best available support. Whilst the use of conventional zygomatic implants is possible in this clinical approach, the use of zygomatic implants manufactured specifically for use in maxillectomy situations possess some advantages. The zygomatic oncology implant (Southern Implants Ltd, South Africa) (Fig. 1) has a 20-mm threaded apical portion for engagement in the zygoma bone with the rest of the implant having a polished surface where it extends into the maxillectomy cavity. This improves the patient’s ability to clean the implant and the defect and reduces the adherence of nasal secretions and food debris. The 55° angulated implant platform head also facilitates screwdriver access and brings it directly into the line of the prosthodontic arch.

The characteristics of a good obturator will improve swallowing, speech function, minimise nasal fluid leakage from the antrum and nasal spaces, restore facial aesthetics including the teeth and facilitate masticatory function and speech. A surgical obturator can be provided at the time of surgery to facilitate function and haemostasis in the immediate post-operative period, and this can subsequently be replaced with a more definitive prosthesis once the maxillary defect has healed to a more stable condition.

To date, no literature exists on the fabrication of an implant-retained maxillary obturator for a paediatric patient, and this case presentation describes the use of zygomatic oncology implants together with the rationale for their use in a paediatric patient.

## Case presentation

**Fig. 2:**
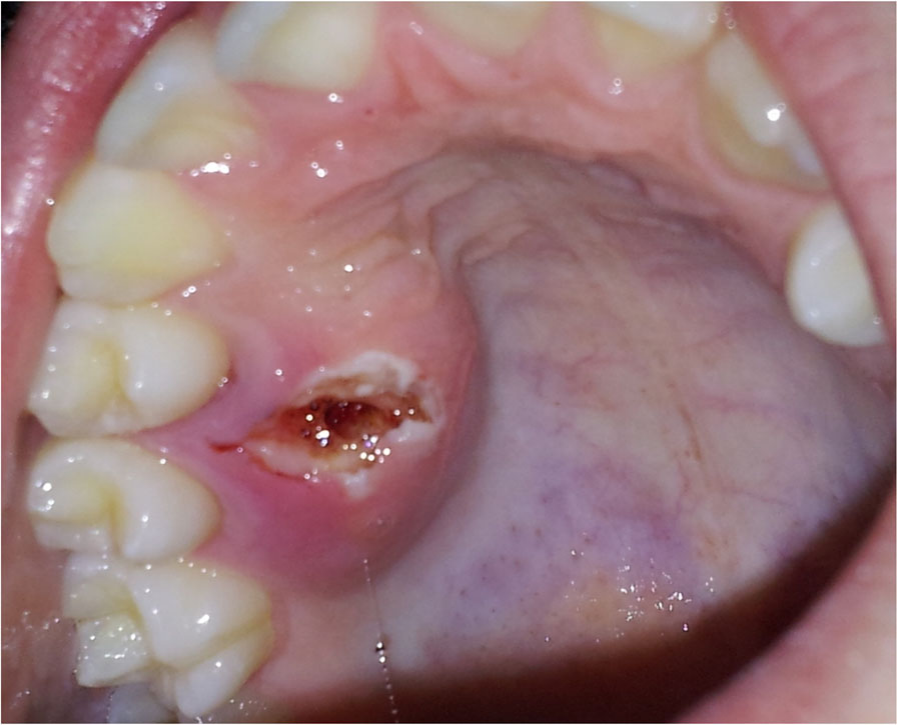
Palatal swelling (post-biopsy) between upper right first and second premolar teeth

A medically fit and well 13-year 11-month-old male was referred to the oral and maxillofacial surgery department at Alder Hey Children’s Hospital in Liverpool in regard to an intra-oral swelling of the right palatal region (Fig. 2). An incisional biopsy was initially reported as a pleomorphic adenoma of the premolar region. Subsequently, a CT scan showed no significant bony abnormality, and a wide local excision was carried out with the application of a surgical palatal dressing plate. Histopathology of this resected tissue appeared to show tumour of intermediate malignant grade at the base of the specimen.

Further investigations undertaken to stage the tumour included a repeat CT scan which presented no evidence of significant bony involvement or erosion. An MRI scan showed no significant asymmetry or signal abnormality in the region of the hard palate, and there was no evidence of loco-regional metastasis of this tumour.

Following a discussion of the craniofacial multidisciplinary team and numerous paediatric pathologists, a diagnosis of intermediate-grade sarcoma of the oral mucosa and hard palate was re-affirmed. A partial right-sided maxillectomy was planned to gain adequate tumour clearance, and prior to surgery, the patient attended for dental impressions and counselling regarding the procedures involved, together with instructions regarding the obturator prosthesis.

**Fig. 3:**
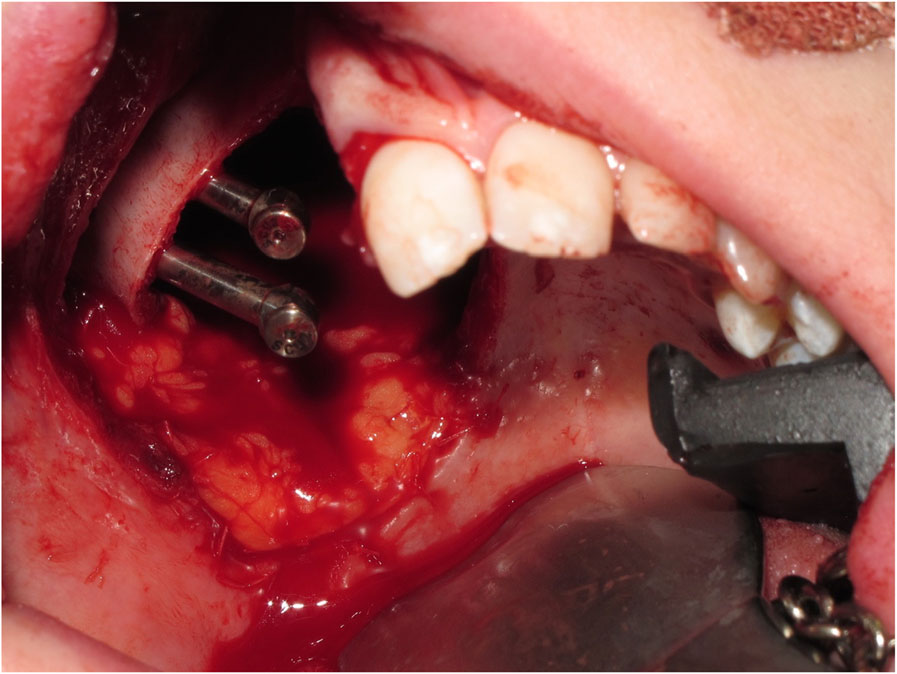
Low-level right-sided maxillectomy with the insertion of two zygomatic oncology implants at time of surgery

A low-level right-sided standard hemi-maxillectomy was carried out via an intra-oral approach with preservation of the pterygoid plates (December 2013). The anterior alveolar cut was undertaken through the right lateral incisor socket following the extraction of this tooth in order to maximise the bone support on the maxillary central incisor abutment tooth. The residual zygomatic body on the right side was exposed, and two 37-mm zygomatic oncology implants (Southern Implants Ltd, South Africa) (Fig. 3) were placed with excellent stability, ensuring that the prosthetic heads were positioned beneath the body of the obturator prosthesis and in a useful position for retention of the obturator. The posterior aspect of the cavity was dressed using the buccal pad of fat and the right inferior turbinate removed to facilitate access to the defect for the obturator. An interim prosthetic obturator was fitted and relined with silicone putty material and retained by dental clasps and a single bone screw into the midline of the remaining palatal bone. Recovery from the procedure was uneventful, and the patient was discharged home the following day. Histopathology confirmed the diagnosis of myxoid spindle cell carcinoma of the right maxilla excised with good margins with no need for adjuvant treatment.

Four weeks later, the patient was returned to the operating room for removal and modification of the obturator. The cavity was healing well, and both implants were firm with no evidence of infection. The initial obturator was modified with the application of a soft lining material and the patient subsequently discharged with instructions on the insertion and removal of the obturator.

**Fig. 4:**
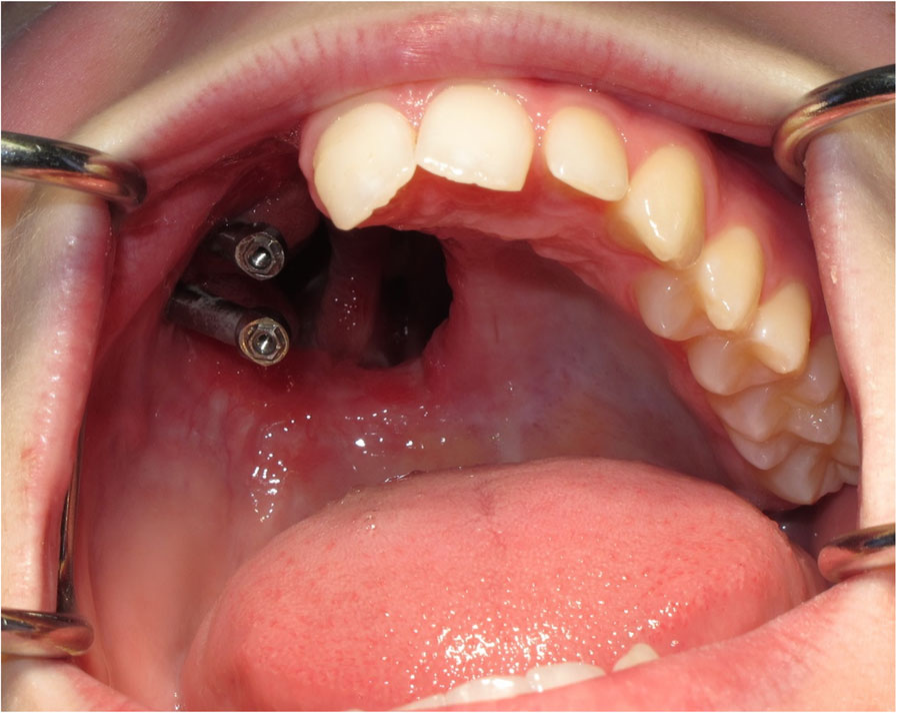
Twelve-week review post-surgery prior to definitive impressions for the implant-supported prosthesis

At the 12-week review (Fig. 4), it was noted the patient had a degree of mucosal polypoidosis in the antral cavity, most probably plaque induced, where the patient found it difficult to clean around the implants. Oral hygiene instruction was reiterated, and construction of the definitive implant bar-retained obturator was commenced.

**Fig. 5:**
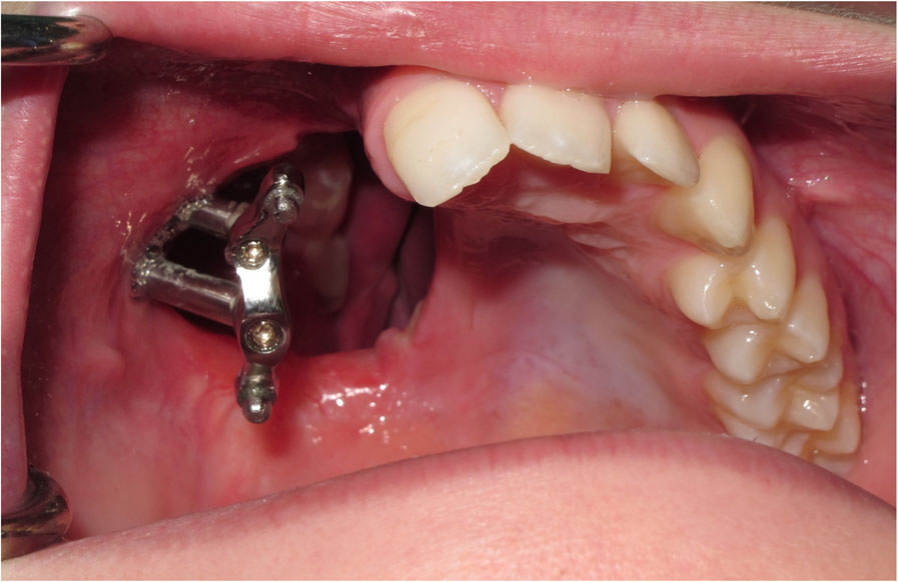
Zygomatic implant bar utilising Rhein attachments for retention

**Fig. 6:**
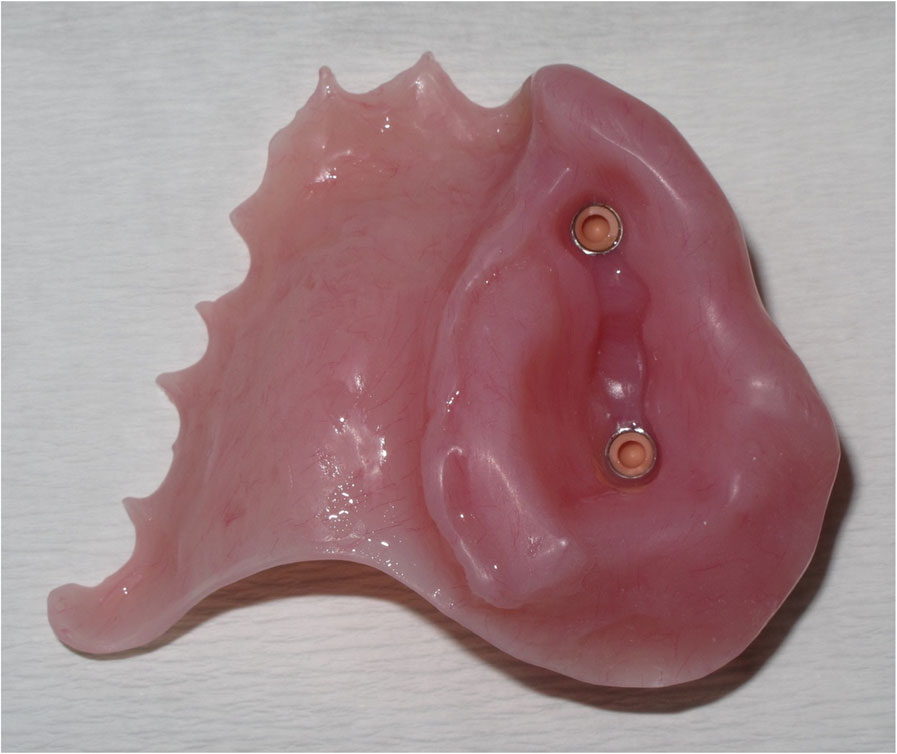
Intaglio surface of definitive acrylic obturator with bar attachments in place. Note the absence of any other retaining clasps and the simple nature of this prosthesis

**Fig. 7:**
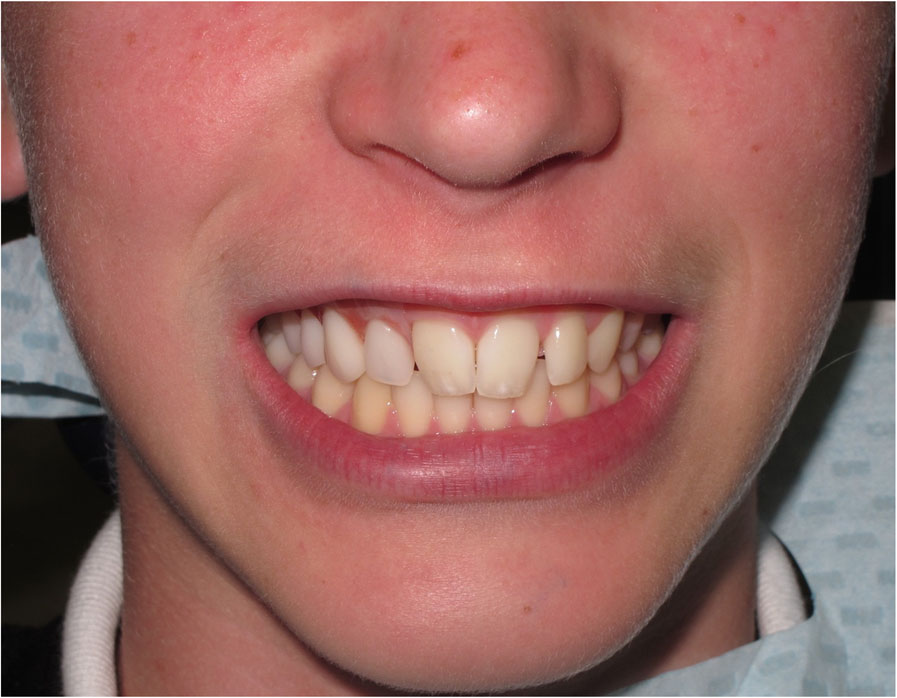
Smile view of definitive implant-retained obturator at initial fitting (April 2014)

**Fig. 8:**
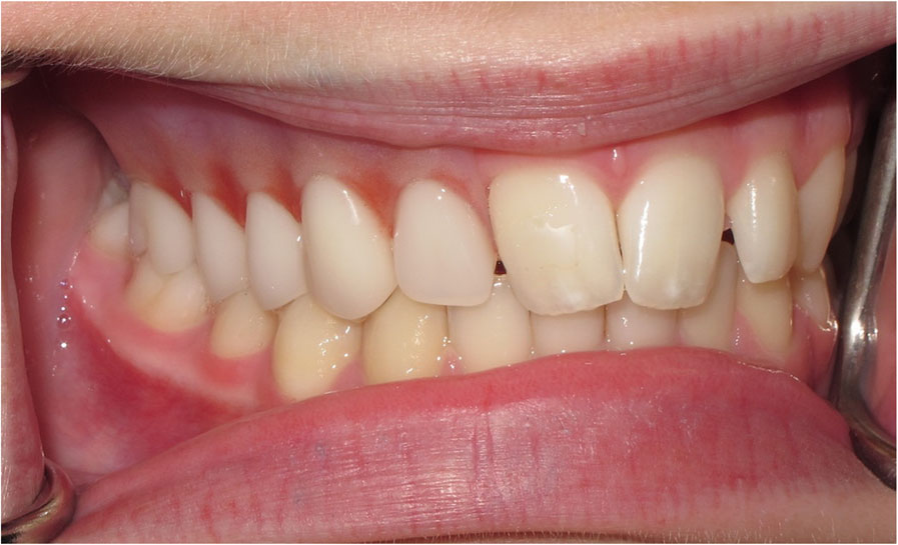
Anterior view of definitive obturator prosthesis in occlusion

**Fig. 9:**
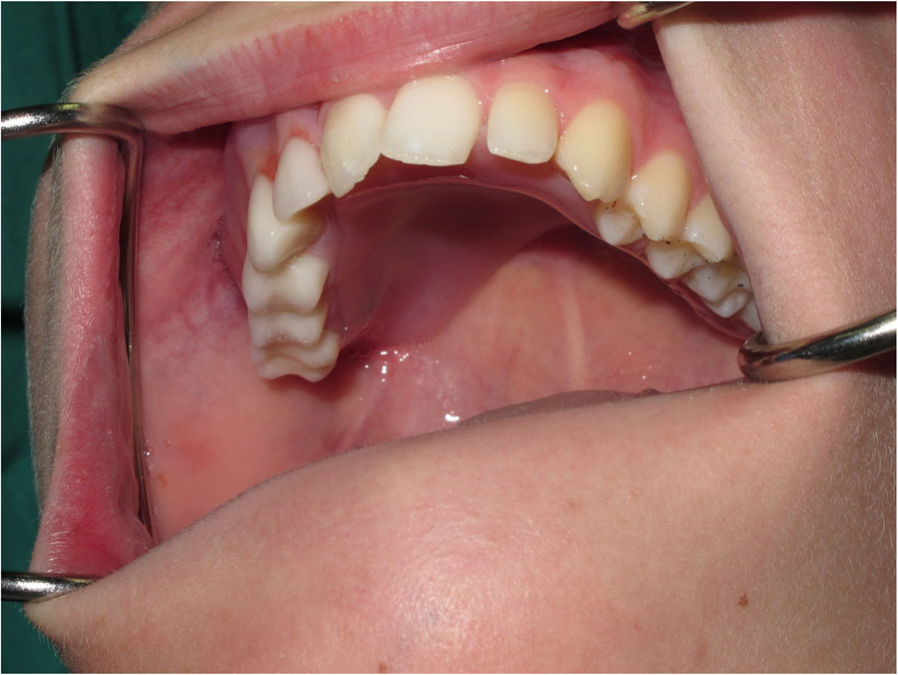
Palatal view of definitive implant-retained obturator at initial fitting (April 2014)

**Fig. 10:**
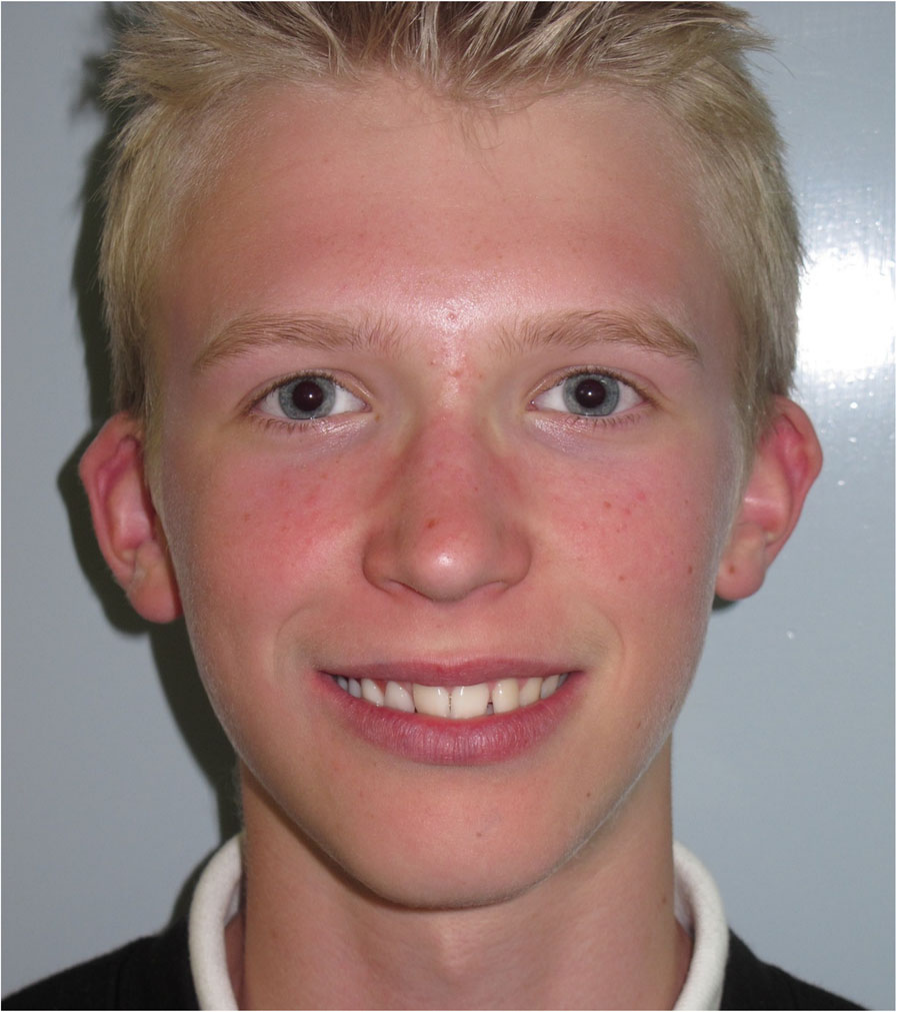
Full facial view of definitive implant-retained obturator at initial fitting (April 2014)

**Fig. 11:**
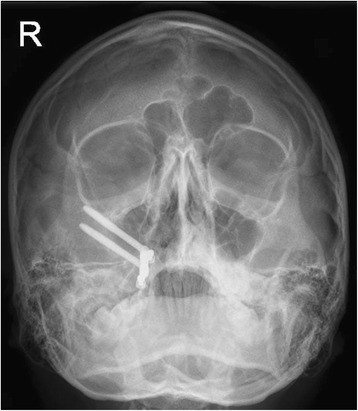
Facial radiograph at 22-month follow-up

**Fig. 12:**
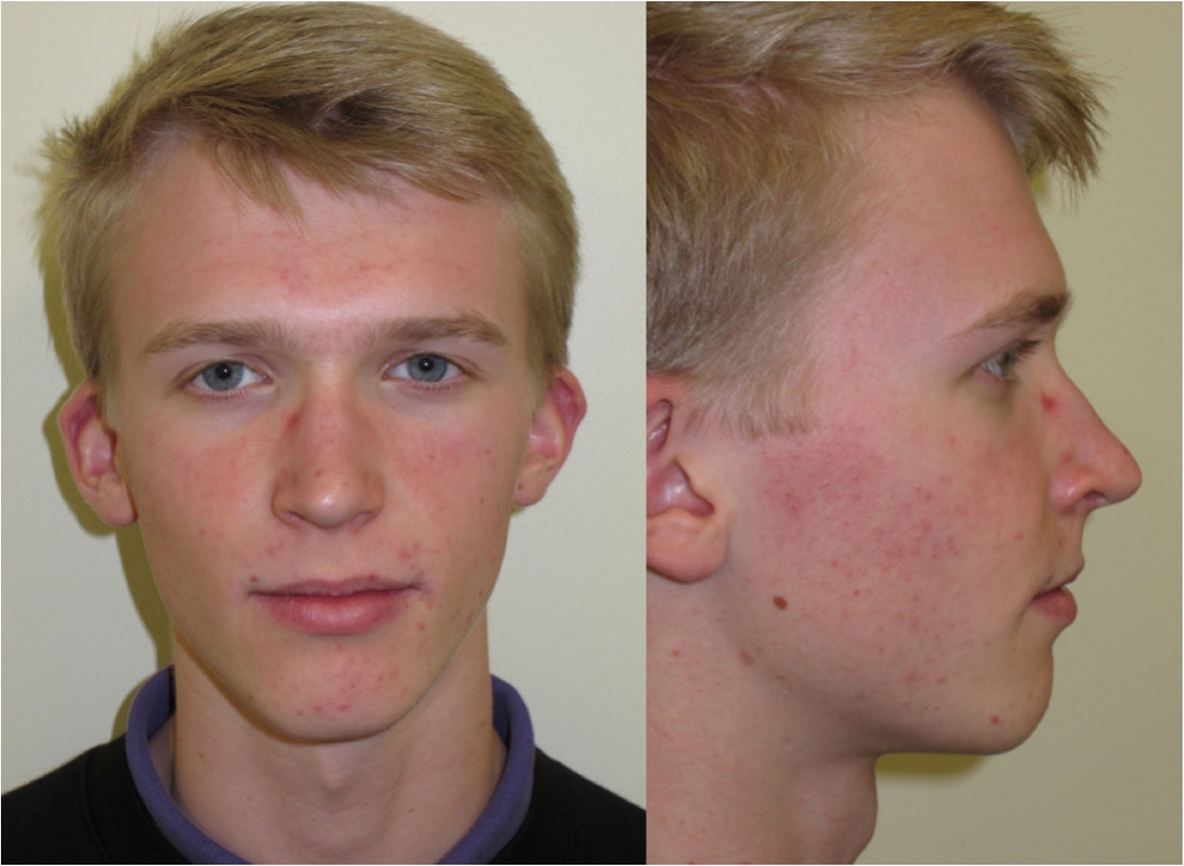
Facial photograph views at 22-month follow-up

Four months following surgery (April 2014), a definitive implant bar-retained maxillary obturator was fitted utilising precision attachments (Rhein attachments, Rhein83, NY, USA.) (Figs. 5 and 6). The retention and support given by the obturator was excellent, and the patient and parents were very pleased with the aesthetic and functional outcome (Figs. 7, 8, 9 and 10) provided by this prosthetic rehabilitation. The patient was put on a regular maintenance programme of review at 6-month intervals and continued to display an excellent standard of oral hygiene around the implants and to report a high degree of oral functioning using it. All mucosal polyposis resolved very quickly following the patient’s improved hygiene measures. He continued under review with no evidence of recurrence or problems with the implants or prosthesis in the 22 months since the surgery. The plastic Rhein female attachments were replaced at 18 months, but no other modifications have been required to this obturator since it was fitted. A recent radiographic review (Fig. 11) demonstrated no significant peri-implant bone resorption, and clinically, there had been no alteration in facial growth or appearance (Fig. 12) of this young patient who was 16 years of age at the time of his final review (February 2017). He continues under regular review.

## Discussion

The paediatric population rarely suffer malignant disease of the oral cavity requiring any form of maxillectomy, and there is little published evidence around the rehabilitation and restorative management of children undergoing such procedures. The seemingly most common approach for a limited low-level maxillary resection in a child would be to consider resection and simple prosthetic obturation as this allows relatively simple management of the tumour from a surgical point of view as well as immediate functional and aesthetic rehabilitation with a prosthesis. It also allows for full histopathological examination of the resected specimen to ensure complete resection of the tumour before committing the patient to any form of complex surgical reconstruction which could be planned at a later date should the patient wish. The delivery of a maxillary obturator improves quality of life significantly by primarily restoring aesthetic and functional modalities. It also serves as a purpose to allow correct phonation of speech, prevent nasal discharge of masticatory contents and facilitate swallowing. The aesthetic and psychological benefits of facial restoration are paramount in a child undergoing such a procedure. The use of microvascular reconstruction techniques have allowed for autogenous tissue reconstruction of maxillary defects with either soft or hard tissue. The use of a soft-tissue-only reconstruction such as a radial forearm flap in this clinical situation would prevent successful dental rehabilitation as the soft tissue flap provides no support for the dental prosthesis and, apart from the separation of the oral and nasal cavities, provides no advantage for the patient. The use of a composite-bone-containing flap such as the fibula flap has the potential to provide oro-nasal separation as well as bone to support an implant-retained prosthesis, and with the latest digital technologies, this can be provided rapidly in carefully selected cases [8], although this mode of rapid rehabilitation is not available in many centres. However, there is no published data on this mode of dental rehabilitation in a growing child currently, and this approach should probably be deferred until all mandibular growth has been completed. The use of microvascular reconstruction, in addition, carries with it significant clinical risks as well as potential donor site morbidity and flap failure as well as the potential for fibrous union and loss of individual bony segments where multiple osteotomies are required.

The difficulty of restoration with a maxillary obturator prosthesis depends on the extent of the surgical resection, with the acceptance that resections with an increasing horizontal component provide a much greater prosthodontic challenge. The number of remaining teeth is a key component in conventional obturator design [9] with the remaining dentition being used exclusively to retain the prosthesis by means of clasps which are often in the visual field and affect the resulting aesthetic outcome for the patient as well as placing significant forces onto them. In conventional maxillary defect preparation, the additional use of a split skin graft into the lateral aspect of the cheek is used to provide a scar band to aid with defect retention, and this brings added morbidity to the procedure especially for a paediatric patient. In conventional hemi-maxillectomy cases, gaining some form of retention from the defect is essential in providing the patient with confidence in the use of the prosthesis, and the discomfort associated with this can make paediatric patients anxious about placing and removing the obturator themselves. The advantages of providing “in-defect” support and retention by means of zygomatic implant placement addresses all of these potential difficulties and allows the construction of a simple highly polished acrylic prosthesis that does not require clasping of the teeth in the aesthetic zone, requires very little extension into the defect to affect a peripheral seal and most of all provides good support when the patient masticates on the defect side. No additional skin grafting is required, and the placement and retrieval of the prosthesis is comfortable and atraumatic. Maintenance of the prosthesis is simple with modifications to the peripheral seal as required at the chair side and replacement of the bar attachments from time to time.

In a paediatric patient, the development and subsequent growth of facial skeleton is an added concern, although by age 13/14, the major mid-face growth will be largely completed [10]. Certainly in this case, the bone volume of the zygomatic body was more than adequate for the placement of the implants. In terms of ongoing facial growth, Min Kim et al. report a case where an 11-year-old male underwent a hemi-maxillectomy and a modified functional obturator (MFO) prosthesis was successfully used to obturate the defect and restore aesthetics and function. After 18 months of wearing the MFO, the result was stable, and at 3 years post-operatively, the patient’s facial profile was reported as near normal. In the case reported here, it was felt that due to the removable nature of the obturator prosthesis, the implant technique employed would allow for the construction of a new maxillary obturator in the event of any significant continued mid-facial/maxillary growth, which so far has not been required.

The use of modified zygomatic implants (Fig. 1) allows improved hygiene by the patient of the implants within the maxillary defect. The threaded portion of the implants is fully engaged into the bone with only the smooth portion protruding into the defect. Clearly this ongoing hygiene by the patient is of utmost importance in preventing peri-implant soft and hard tissue changes, but the implant design here has facilitated that extremely well in this young patient, and no additional professionally directed hygiene measures to maintain excellent peri-implant health have been required to date.

The evidence for loading zygomatic implants immediately is very clear in the literature [11] in a cross-arch manner but not in a unilateral situation as has been utilised in this case. Whilst the stability of the implants achieved at surgery was excellent, it was decided to adopt a delayed loading approach which also gave time for the maxillectomy defect to stabilise prior to the construction of the definitive prosthesis.

## Conclusions

The use of zygomatic implants to supplement the stability and retention of the maxillary obturator in this case has improved the function of the prosthesis and provided for a very high-quality rehabilitation for the patient reported with no evidence of disruption to facial growth in the 22 months following surgery.
